# Bilateral Frontal Sinus and Epidural Mucopyocele Drainage Using “Modified” Draf III

**DOI:** 10.1155/crot/7636938

**Published:** 2025-09-04

**Authors:** Shahrokh Khoshsirat, Mohammad Samadian, Afsoon Zandi, Ilia Mirzaei, Seyed Taher Mousavian

**Affiliations:** ^1^Department of Otolaryngology, Head and Neck Surgery, Loghman Hakim Hospital, Shahid Beheshti University of Medical Sciences, Tehran, Iran; ^2^Department of Otolaryngology, Hearing Disorders Research Center, Loghman Hakim Hospital, Shahid Beheshti University of Medical Sciences, Tehran, Iran; ^3^Department of Neurosurgery, Skull Base Research Center, Loghman Hakim Medical Center, Shahid Beheshti University of Medical Sciences, Tehran, Iran; ^4^Department of Otorhinolaryngology, Masih Daneshvari Hospital, Shahid Beheshti University of Medical Sciences, Tehran, Iran; ^5^Neurosurgery Department, Sina Trauma and Surgery Research Center, Tehran University of Medical Sciences, Tehran, Iran; ^6^Hearing Disorders Research Center, Loghman Hakim Hospital, Shahid Beheshti University of Medical Sciences, Tehran, Iran

**Keywords:** Draf III, frontal epidural abscess, mucopyocele drainage, trephination

## Abstract

**Introduction:** Draf III is a surgical technique which uses endoscopic approach to access the frontal sinus, by leaving almost no skin markings and having minimal side effects. The technique is most frequently indicated for frontal chronic refractory sinusitis, followed by mucoceles and skull base or paranasal tumors. The aim of Draf III is to drain frontal sinus masses and collections.

**Clinical Presentation:** In this report, we present a 16-year-old male with progressive headaches, dizziness, nausea, and vomiting whose imaging revealed left-predominant bilateral frontal epidural mucocele and abscess. A Draf III approach was chosen, to which bilateral supraorbital trephinations were added for better drainage of the collection. Mucosal grafts were used to avoid crusting and stenosis. Post-operation included a course of antibiotics and steroids with three sessions. On the twelfth-month follow-up, the patient was doing well and had no complaints.

**Conclusion:** A modified Draf III approach is effective for the management of bilateral frontal mucopyocele in treatment-resistant cases, without causing any major complications.

## 1. Introduction

Draf III is a challenging endoscopic approach aimed at the removal of the superior part of the nasal septum, the frontal beak, and the interfrontal septum to create a common frontal sinus cavity. It is most commonly used in frontal chronic refractory rhinosinusitis, followed by mucoceles, skull-based or paranasal tumors, or Draf IIa failure. Wolfgang Draf described a series of procedures in 1991 to open the frontal sinus outflow tract more widely, to address the difficulty of endoscopic frontal sinus surgery, the most extensive of which is Draf III [1–3]. The Draf classification includes four procedures, each progressively more extensive. Draf I—the least extensive—opens the frontal recess by removing the ethmoid bulla and other ethmoidal cells obstructing the outflow tract. Draf IIa expands the frontal recess by removing the agger nasi cells and ethmoidal bulla completely, up to the middle turbinate. Draf IIb extends the recess medially to the nasal and intersinus septum. Draf III is by extension, a bilateral Draf IIb, combined with a superior septectomy and removal of intersinus septation to create a common frontal sinus cavity [2–5].

Charles Gross further modified the procedure in 1995 to its current state called the endoscopic modified Lothrop procedure [2, 5, 6]. According to Shih et al. and Chiari et al., Draf III is used to manage benign frontal sinus masses like mucoceles, which was the approach of choice in 10.5%–11% of frontal mucoceles [7, 8]. Limitations of Draf III can be noted in a short anteroposterior frontal recess, which reduces the success rate, leading to direct skin manipulation [9]. Frontal sinus trephination is an opening into the frontal sinus via supraorbital incisions beneath the eyebrows. It can be used either in tandem with intranasal endoscopic approaches for an “above and below” access to the frontal sinus, i.e., accessing the portions of the frontal sinus unavailable to via an endoscope, or used in recurrent acute frontal sinusitis that is refractory to other medical therapies [10, 11]. More significant to the purposes of this report is the access it provides for further irrigation, which is most useful in acute and recurrent mucopurulence [12].

Frontal sinusitis can be complicated by paranasal sinusitis, osteomyelitis, or Pott's puffy tumor, yet in most cases, an exact etiology is unknown [13].

In this report, we present a case of a 16-year-old boy with progressive headaches, dizziness, and nausea whose bilateral frontal sinus mucopyoceles and abscess were drained via Draf III along with trephination of the frontal sinuses for better access and drainage, as well as checking for the post-drainage mucosal and frontal sinus integrity.

## 2. Case Presentation

**Figure 1:**
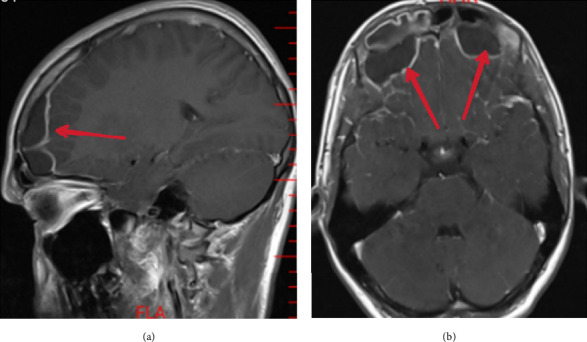
The brain MRI of the patient. (a) A sagittal T1-weighted view of the brain, showing 1.1 cm collection of fluids in the frontal sinus. (b) An axial T1-weighted view of the brain which shows bilateral frontal epidural mucocele. Note the bigger size of the collection on the left side. The frontal sinus collection extends toward the cranium which is seen as an epidural mucopyocele. The dura appears intact, but the posterior wall of the frontal sinus and the corresponding epidural space were damaged, both of which were confirmed by viewing the CT scan performed at the patient's hometown hospital and noted during the operation.

The patient was a 16-year-old boy, who had developed a progressively worsening headache, dizziness, nausea, and vomiting over 2 weeks. He had no significant previous medical histories and had no other symptoms prior to those he had come in with. He was initially treated palliatively by his general practitioner on his 3^rd^ day of symptoms, but given a lack of improvement in addition to the growth of a small bulging that appeared on his forehead (above his eyebrows, about 2 cm in diameter), he was admitted to a local hospital, during which a head CT scan showed a collection in the frontal sinus with damaged posterior wall extending into the epidural space. The patient was referred to Day-Hospital in Tehran for neurosurgical consultation and underwent magnetic resonance imaging (MRI) scanning [4, 14]. A repeat CT scan was not performed as the patient had brought the imaging from his hometown hospital. Upon entry, routine laboratory tests were performed which showed leukocytosis (14.4 × 1000/μlit) and elevated erythrocyte sedimentation rate (ESR, 50 mm/hr). (Figure 1) The MRI scan (Figure 1) confirmed the fluid as bilateral purulent-filled anterior frontal lobes of the brain, situated behind the posterior table of the frontal bone, indicating its localization within the frontal epidural. . Therefore, a diagnosis of bilateral frontal epidural mucocele was established, and the patient was sent to the operating room for surgical drainage via “modified” Draf III.

### 2.1. Surgical Procedure

An inside-out Draf III was planned to drain the mucocele through the frontal sinus. Furthermore, we incorporated the trephination of the frontal sinus into the procedure; two bilateral supraorbital incisions, beneath the eyebrows, allowed for better access to the frontal sinus and facilitated drainage of the mucopyocele.

**Figure 2:**
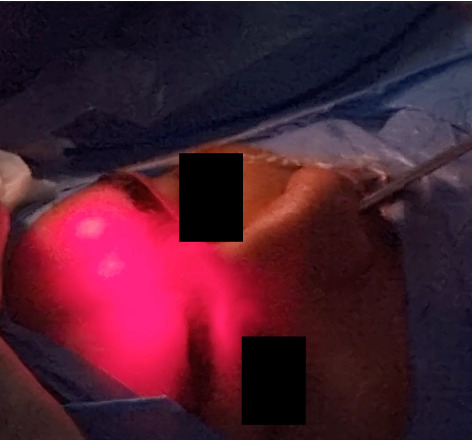
Transillumination of the frontal sinus, showing intact sinuses without obstructions. This indicates a correct entry and direct access to the sinus from the nasal septum. The patient's consent was obtained before the surgery, for both the use of picture and report.

The procedure began by drilling the frontal beak and removing the uncinate process and connecting bridge between the lacrimal bone's superior and posterior portions using a 30° endoscope. Then, the anterior and medial agger nasi cells were removed using a 2-mm Kerrison punch, and the frontal recess was widened using angled forceps. The floor of the frontal sinus was identified bilaterally, and a 2 cm window was opened into each side, extending to the middle turbinate and connecting the two frontal sinuses. Prior to drilling the window, the boundaries were identified and marked using a Medium size fine tip dissector. Then two access points to the frontal sinus were made: (1) a 4-5 mm access via the nasal cavity and (2) bilateral 5 mm trephinations of the skin (beneath the eyebrows) [2, 4, 15]. The trephination of the frontal sinus was our addition to the Draf III procedure, which allows for better access, more thorough drainage, and suction of the mucocele within the frontal sinus. The frontal recess was drilled from the outside, in an anterolateral direction into the frontal sinus toward the contralateral sinus. A 30° 3 mm cutting burr was used to remove the dense bones, including the entire frontal beak, frontal sinus floor, and the bony septum. Transillumination revealed the frontal sinus in their entirety (Figure 2). The established access to the frontal sinus allowed for a successful mucocele drainage. The trephination allowed for further suctioning of the deeper parts of the frontal sinus as well as the portions of the mucocele that were not drained via the inferior window of the frontal sinus. To finish the procedure, the skin incisions beneath the eyebrows were closed, and the anatomic landmarks of the nasal cavity were checked. To avoid crusting and stenosis in the future, a mucosal flap was inserted over the exposed posterior bone (posterior inferior turbinate flap).

### 2.2. Post-Operation Care

Post-operative management included 500mg of Metronidazole twice a day, 1g of Cefotaxime twice a day, and a 20mg Dexamethasone injection. Given that an abscess was formed as a complication, an infectious consult was requested to start vancomycin and imipenem should they be necessary. Nasal saline wash was taught and ordered to be started within 1 week after the operation. Follow-up appointments were scheduled at1-week, 1-month, 3-months, 6-months, and 12-monthsfollowing the procedure . In the final (12-month) follow-up visit, the patient was doing well and had no complications and the endoscopic transillumination revealed a patent common frontal sinus cavity. As no symptoms were present and no surgical complications were observed, further imaging was not performed in the follow-ups.

## 3. Discussion

This case report included a Draf III with the trephination of the frontal sinus via bilateral supraorbital incisions to drain an epidural mucocele.

Rhinogenic headaches develop due to either nasal sinusitis or abnormal anatomic variation of the sinus. Those with a sinusitis origin can either be categorized into inflammatory and noninflammatory or acute and chronic all of which can lead to secondary episodes of headaches [16]. Given that the development of frontal sinus is as an outpouching from the frontal recess growing into the frontal bone and continues until the age of 16–18, infections from frontal sinus can seep intracranially via the valveless diploic veins [13]. We consider the distinction between intracranial and frontal abscess formation. Notably, cases of Rhinogenic occipital abscess formation have been reported. Madala et al.'s presentation offers an intriguing account of such complications, characterized by headaches, fever, and neck stiffness. [17]. Our patient had progressive headaches, dizziness, nausea, and vomiting, the symptoms seen in increased intracranial pressures, due to the mucocele in the frontal sinus.

As with any intracranial complications, the basis of the diagnosis is imaging, whether via thin-sliced CT scans or MRI of the brain [16–19]. The current advances in neuroimaging have made prompt diagnosis possible, while laboratory panels help with identifying the extent of complication. In addition to his symptoms, our patient had a leukocytosis of 14,400 and an ESR of 50 both of which were above the normal range, indicating an inflammatory status besides his mucocele, or better said, an abscess.

An inside-out Draf III approach is the standard Draf III procedure which widens the frontal recess by drilling through it in an anteromedial direction, allowing for an easier access to the frontal sinus. The inflammatory nature of the case and an absence of anatomical abnormalities that contraindicate the procedure, i.e., an anteroposterior frontal recess narrower than 5 mm, made this approach a more reasonable one. In contrast, an outside-in approach is chosen when the frontal recess is narrow. Both approaches are identical up until drilling, which in the outside-in approach begins with the drilling of frontal beak instead of the frontal recess and then includes the frontal sinus and intersinus septum, followed by the lateral expansion of neostium to include the frontal recess [2, 4, 15, 20]. Chin et al. published a case-control study of 30 patients on whom they performed an outside-in modified endoscopic Lothrop procedure (MELP). An outside-in procedure is chosen when inflammation or tumor involves the frontal recess, addressing the issue by starting the sinusotomy first and moving toward the frontal recess. By describing the outside-in procedure in detail, the authors recorded the perioperative complications and the operative time, concluding the technical feasibility achieved by a wide access to the frontal recess and efficient development of the Lothrop cavity. However, in our case, while an outside-in approach seemed like a more feasible choice, the inside-out approach was chosen due to the wider frontal recess of our patient to accommodate 4-5 mm instrumental access and to implement the trephinations later on in the procedure. Advantages to an outside-in procedure include its applicability to an occupied frontal recess that limits drilling and minimal mucosal bleeding. However, there is a concern for skull base injury especially in cases with difficulties identifying the olfactory fascicles and the created posterior edge of the septal window [2, 15].

Trephination of the frontal sinus is indicated with extra-sinus spread of the frontal sinusitis, osteomyelitis of the sinus (e.g., Pott's puffy tumor), lateral lesions of the frontal sinus, fibro-osseous tumor resections, and soft tissue tumors [21]. In combination, these two methods provide full access to the frontal sinus for complete drainage of the mucocele without any complications.

Frontal sinus complications can be categorized into three subgroups based on their etiologies: infection, trauma, and surgical. Infectious frontal sinus complications are rare but can occur secondary to acute rhinosinusitis. Acute sinusitis is a mild condition with rare complications, which most commonly extend toward the eye and the cranium. Cranial extensions can cause meningitis, cerebritis, epidural abscess, subdural empyema, and even brain abscesses, all of which have significant morbidities including strokes and seizures. Otto et al. described suppurative frontal sinus complications in pediatrics and reported 78% headaches and 39% nausea and vomiting [22]. 56% of their patients had congestions as well, but our patient had no nasal symptoms. Yang et al. reported a frontal sinus abscess in a 14-year-old boy which was complicated by an epidural abscess. Their patient had fever, headache, and dizziness, while our patient had those along with nausea and vomiting. They performed an open drainage of the maxillary sinus and frontal sinus under general anesthesia with nasal endoscopy assistance. However, given the lack of maxillary involvement in our case, we only had to focus on frontal sinus drainage, thus our decision on Draf III procedure [23]. Al Yaeesh et al. reported 5 cases of frontal sinus complications including an orbital abscess, meningitis with superior sagittal sinus thrombosis, subdural empyema, frontal lobe abscess, and frontal bone osteomyelitis with frontocutaneous fistula. Their most relevant case to ours is their frontal lobe abscess which was managed with endoscopic frontal drainage and frontal craniotomy. Given the lack of a cerebral abscess in our case, we did not need to perform a craniotomy. In our case, the absence of a cerebral abscess necessitated the avoidance of a craniotomy. Nevertheless, the presence of a mucocele within the frontal sinus and the epidural involvement necessitated Draf III, along with trephination within the frontal sinus, for the complete drainage of the mucocele. [24].

Other surgical methods within this category fall into the combined frontal sinus surgical approaches. These include combined endonasal-transorbital approach to manage the far lateral frontal sinus, rhino-frontal sinuseptotomy (RFS), combined endoscopic and intraoral approach or endoscopic approach alone for the management of odontogenic sinusitis, and transnasal endoscopic and combined intra–extranasal approach for the surgical treatment of traumatic frontal sinus cerebrospinal fluid rhinorrhea [25–27]. As the nomenclature suggests, the most relevant of the approaches to our case is the RFS. First introduced by Eberhard Stennert in 2001, this approach is indicated for cases resistant to surgical and medical treatments, recurrent phlegmons of the facial skin or the orbit, and mucopyoceles of the frontal sinus. In comparison, Draf III with trephinations beneath the eyebrows is a less invasive approach with fewer complications [28]. Makary et al. reported a combined frontal sinus approach for lateral frontal sinus diseases with orbital extensions. Due to the orbital extension of the cases, the authors reported they added an upper lid crease approach to all their procedures, which they reported as Draf IIa and Draf IIb. The authors performed no Draf III, but all their cases included mucopyoceles like ours. We did not face an orbital involvement in our case; thus, lateralization of the frontal sinuses was not the focus of our approach, as much as draining them was. The upper lid crease incisions of their approach heal with minimal cosmetic issues similar to our trephinations. Despite the similarities in both approaches, Draf III seems more suitable in cases closer to intracranial involvement (e.g., epidural abscess) and Draf IIa and IIb seem better when lateralization is required (e.g., orbital involvements) [29].

With modern advancements in endoscopic sinus surgeries (ESSs), more invasive external surgeries, such as osteoplastic flaps (OPLs) and neurosurgical routes like craniotomies, are reserved for when ESS is not suitable or has failed as these procedures are associated with significant morbidities. For conditions like extensive diseases, frontal sinus tumors, and osteomyelitis that require better visualization of the complex frontal recess anatomy, an open approach seems more suitable [30]. Given the accessibility of the mucopyocele in our case via an endoscopic route, its anteromedial distribution without much lateralization, and the size, minimally invasive endoscopic approach seemed better in comparison to an open approach.

A limitation of this case report is the lack of inclusion of the preoperative CT scan performed at the patient's hometown hospital, which was unrecoverable post-discharge due to data access constraints.

In conclusion, Draf III is an appropriate approach to draining the frontal sinus mucocele and abscess with an easier access to the frontal sinus. With little to no complications, and minimal side effects, patients indicated for the procedure can be safely treated and followed.

## Data Availability

The data that support the findings of this study are available on request from the corresponding author. The data are not publicly available due to privacy or ethical restrictions.
